# Does Bacteria Colonization of Canine Newborns Start in the Uterus?

**DOI:** 10.3390/ani11051415

**Published:** 2021-05-14

**Authors:** Ada Rota, Andrea Del Carro, Alessia Bertero, Angela Del Carro, Alessandro Starvaggi Cucuzza, Penelope Banchi, Michela Corrò

**Affiliations:** 1 Department of Veterinary Sciences, University of Turin, 10095 Grugliasco, Italy; angela.delcarro@unito.it (A.D.C.); alessandro.starvaggi@unito.it (A.S.C.); penelope.banchi@unito.it (P.B.); 2 Iunovet-Clinique Vetérinaire Saint Hubert, 06240 Beausoleil, France; andrea.delcarro@gmail.com; 3 Veterinary Practitioner, 10126 Turin, Italy; alessia.bertero@gmail.com; 4 Istituto Zooprofilattico Sperimentale delle Venezie, 35020 Legnaro, Italy; mcorro@izsvenezie.it

**Keywords:** dog, pregnancy, bacterial flora, fetus, amniotic fluid, meconium

## Abstract

**Simple Summary:**

A well-balanced microbial flora plays a fundamental role in puppies’ early development. Bacteria were thought to colonize newborns at birth, but some studies have challenged this hypothesis. A healthy fetus at term may already harbour bacteria and the uterus may also not be sterile. Time of initial microbiota development might be placed earlier in life. In this investigation we sampled uterus, amniotic fluid and meconium of healthy canine fetuses delivered through cesarean section (elective or emergency) and carried out bacteriological examinations. In contrast with the ‘sterile womb paradigm’, bacteria were isolated from all the sampled sites and materials, independently of the cesarean type. Further studies are necessary to confirm our results. We adopted traditional bacteria culture techniques, but molecular methods, which look for bacteria DNA, could also be performed to deepen the knowledge on this matter.

**Abstract:**

The assumption that requires the uterus to be a sterile environment to sustain a successful pregnancy has been recently challenged in humans, and is still under debate. The aim of this study was to assess whether bacteria can be isolated from the pregnant uterus and from amniotic fluid and meconium of healthy canine fetuses at term, delivered through cesarean section. Fifteen dams of different breed, age and parity, undergoing either elective (*n* = 10) or emergency (*n* = 5) cesarean section after a healthy pregnancy, were included in the study. Swabs for bacterial culture were collected from the uterus, and from amniotic fluid and meconium. Bacteria were isolated from all the sampled sites and materials, irrespective of cesarean type. In most cases, different bacteria were isolated from the different sites. *Acinetobacter* spp., coagulase-negative Staphylococci and *Bacillus* spp. were frequently found while *Pseudomonas aeruginosa*, *Micrococcus* spp., *Moraxella* spp., *Macrococcus* spp., *Glutamicibacter* spp., *Stenotrophomonas* spp. and *Psychrobacter* spp. were only occasionally identified. Our data show that uterus and fetuses may not be sterile in healthy term canine pregnancies.

## 1. Introduction

As in human infants, resident microbial communities are thought to play an important role in puppies’ early development, in adaptation to environment and development of immunity. Understanding the timing of the initial microbial colonization could be useful to better understand the features of a well-balanced microbial flora. In children, dysbiosis has been associated to many diseases such as inflammatory bowel diseases, type 1 diabetes, necrotizing enterocolitis, and asthma [1]. Placenta, amniotic fluid and fetus have traditionally been viewed as sterile in healthy human pregnancies. According to the ‘sterile womb paradigm’, bacteria are acquired both from the mother and from the environment during and after birth [2]. However, some studies have suggested the existence of a placental bacterial community [3,4]. Culture and molecular methods detected bacteria in human amniotic fluid samples [2]. More recent works, using molecular-based methods, gave controversial results: some studies discovered that amniotic fluid is sterile in healthy term pregnancies [5,6] while others found bacteria DNA [7]. Also the fetal gut may not be sterile and bacteria have been described in human meconium [7,8]. Vertical transmission is also suggested in experiments that used an in vivo rat model, in which maternally derived *Enterococcus faecium* [8] and bifidobacterial strains [9] were found in the gut of newborns delivered by cesarean section. The hypothesis of an intra-uterine colonization is still under debate and, although an intra-uterine seeding could occur, the importance of prenatal microbial colonization is still to be defined [1,8].

Little is known about the pattern of microbial colonization of canine newborns and about the origin of bacteria. In a recent study, bacteria were isolated from puppies’ placenta and meconium but vaginally delivered puppies were included in the investigation and meconium was sampled after colostrum intake [10].

The aim of this study was to assess whether bacteria can be isolated from the pregnant uterus and from the amniotic fluid and meconium of healthy canine fetuses at term, delivered through cesarean section.

## 2. Materials and Methods

### 2.1. Animals and Samples

The work was approved by the Ethical Committee of the Department of Veterinary Sciences of the University of Turin (Italy) (66/10/01/2020). Written informed consent of the dog owners was obtained. All procedures were performed in compliance with the guidelines of the Italian Ministry of Health for the care and use of animals (D.L. 4 March 2014 n. 26 and D.L. 27 January 1992 n. 116) and with EU Directive 86/609/CEE.

Fifteen dams of different breed, age (3.7 ± 1.8 yrs; mean ± SD), weight (26.8 ± 25.9 Kg) and parity were included in the study (Table 1). They underwent either elective (*n* = 10) or emergency (*n* = 5) cesarean section (CS), at the Veterinary Hospital of the University of Turin (Italy) or at a private practice (Iunovet-Clinique Vetérinaire Saint Hubert, Beausoleil, France), from January to November 2020 (Table 1). All pregnancies had been uneventful and no antimicrobial agents or other drugs had been administered to the animals.

In the periparturient period the animals were monitored for behavioral alteration associated with forthcoming parturition (restlessness, nesting behavior, vomiting). Elective CSs were planned on the bases of the first day of LH surge, indirectly detected through progesterone evaluation. Plasma progesterone was measured daily or every other day starting from the 60th day from the estimated LH surge. Elective CSs were carried out when plasma progesterone concentration was ≤2 ng/mL [11], or, in four cases, when the cervical mucous plug was observed passing the vulva [12]. In a single case (Table 1, Bitch *n* = 8), CS was carried out before the natural decrease of progesterone because of the exceptionally high fetal number, signs of dropsy of the fetal sacs and severe respiratory distress of the dam.

Emergency CSs were done because of uterine inertia or obstruction (Table 1). Before surgery, the bitches underwent clinical examination and routine blood exams; a quick abdominal ultrasound examination was done to check fetal heart rates. The surgical site preparation was carried out as follows: hair clipping was performed outside the operating room to prevent contamination, then for the aseptic scrub two antiseptic solutions (70% ethanol and povidone iodine, three passages each) were applied on the site using gauze sponges. Four surgical drapes (Santex Spa, Sarego, Italia) were used to delimit the operating area.

### 2.2. Bacterial Culture

In all the bitches, inhalatory anesthesia was maintained with isoflurane after induction either with propofol or with alfaxolone, and after preoxygenation. Incision of the uterus was done at the base of a uterine horn.

For sampling, swabs of two different sizes were used. A ‘regular’ swab (ESwab, 480CE, Copan Italia Spa, Brescia, Italia) was collected at the site of placental insertion of the first extracted fetus. A sample of amniotic fluid was collected into a sterile syringe from the first extracted fetus, before membrane rupture: the amniotic fluid was then dropped onto another ‘regular’ swab (ESwab, 480CE, Copan Italia Spa). The fetus was passed to an assistant wearing sterile gloves who dried the puppy with sterile paper towels and, after resuscitation, collected a sample of meconium from the rectum using a ‘mini’ swab (ESwab, 484CE, Copan Italia Spa). A regular and a mini swab were left open on the instrument table until the end of surgery, to act as an environmental control. All swabs were placed into their 5 mL tubes containing 1 mL of modified Liquid Amies medium (ESwab^®^ Copan Italia Spa).

The CSs went on as usual, until the extraction of the last fetus (when 20 mg/kg i.v. cefazolin were administered) and standard uterine and abdominal closure were performed. The samples were transported to the laboratory in refrigerated boxes at 6–8 °C of temperature, and were cultured within 48 h of collection.

Bacterial isolation was performed according to standard lab culture techniques. Briefly, each swab was diluted in 1 mL of nutritive broth (Heart Infusion Broth, HIB, Conda, Madrid, Spain); 10 µL and 100 µL from this suspension were respectively inoculated into different solid and liquid media before incubation in different atmospheric conditions, as follows: (i) search for aerobic microorganisms: nutrient blood agar (Blood Agar Base n° 2, BA, Biolife, Milan, Italy), with 5% sterile defibrinated sheep blood (Allevamento Blood, Teramo, Italy) and the following selective media were inoculated and incubated at 37 °C ± 1 °C for 24 h under aerobic conditions: *Enterobacteriaceae* medium (McConkey agar, Thermo Fisher Oxoid Ltd., Basingstoke, UK) and bile-esculin azide agar, BEA (Conda; (ii) search for anaerobic microorganisms: nutrient blood agar, BA medium, selective medium for *Clostridium perfringens,* PBA (TSC Agar Base with specific *C. perfringens* selective supplement, Biolife) added with 5% sterile defibrinated sheep blood, and fluid thioglycolate medium (Liofilchem, Teramo, Italy) were inoculated. The media were placed in anaerobic jars with anaerobic generating system and anaerobic indicator AnaeroGen 2.5 L/3.5 L/Compact—Atmosphere Generation System and Anaerobic indicator BR0055b (Thermo Fisher Oxoid Ltd.) and incubated at 37 °C ± 1 °C, for 48 h. All inoculation steps were performed quickly to limit the oxygen exposure of the inoculated material.

The culture media were evaluated at 24 or 48 h depending on the aerobic or anaerobic conditions; and in case of absence of bacterial growth on the agar culture media, but turbidity of the nutrient broths, the plates were re-incubated for a further 24 or 48 h respectively, and broth seeding as previously described was performed.

All the microbial colonies grown on the first isolation plates were counted. On the basis of the number of colony forming units (CFUs), growth was classified as Low (1–10 CFU/10 µL), Moderate (11–30 CFU), or High (≥31 CFU).

Genus identification of bacteria was phenotypically performed by macroscopic observation of colonies, Gram stain reaction, cellular morphology, growth on selective medium, catalase, oxidase, mobility tests and coagulase tube test. Species identification was performed by MALDI-TOF MS: Microflex LT instrument (MALDI Biotyper, Bruker Daltonics, Billerica, MA, USA) equipped with FlexControl software (version 3.3, Bruker Daltonics).

## 3. Results

In all the elective CSs, dam clinical parameters were within normal limits and fetal heart rate was above 200 bpm. Also in emergency CSs, the dam parameters were normal, but fetal heart rate was occasionally between 150 and 180 bpm.

A total number of 73 alive puppies were born through elective CSs: two litters each contained an immature fetus and a third one a dead fetus. All the 17 fetuses extracted in emergency CSs were alive; only the naturally delivered fetus of a Dachshund bitch was born dead (Table 1).

Control swabs did not result in bacteria growth. Bacteria were isolated from all the sampled sites and materials, irrespective of Cesarean type (elective or emergency) or surgical facility (Table 2). In elective CSs, bacteria were isolated from six uterine samples out of nine, from five amniotic samples out of ten, and from eight meconium samples out of ten; in emergency CSs, bacteria were isolated from two uterine sample out of four, from one amniotic sample out of five, and from four meconium samples out of five. Only three cases tested negative in all the sampled locations (two elective and an emergency CS); however, two of them were incomplete, lacking uterine samples (Table 2).

Pure culture resulted in four uterine samples, four amniotic fluid samples and six meconium samples. Meconium showed the single case in which three different bacteria were isolated. The number of pure vs. mixed cultures was not significantly different among sampling locations (Fisher’s exact test; *p* = 0.78).

Irrespective of the origin of the sample, bacteria growth was sometimes limited to few colonies, but many times a high or moderate growth was recorded. In the majority of cases, different bacteria were isolated from the different sites.

Bacteria of the genus *Acinetobacter* were isolated in many samples, from eight out of twelve bitches, more frequently in meconium samples (*n* = 8), occasionally in uterine cultures (*n* = 2) or in amniotic fluid (*n* = 1) (Table 3). Species identification led to the detection of *Acinetobacter baumannii* (*A. baumannii*) in four meconium samples. In the majority of cases, *Acineobacter* showed high growth in culture from meconium; only in one bitch *Acinetobacter* spp. was isolated from uterus, amniotic fluid and meconium (Table 3).

*Pseudomonas aeruginosa* (*P. aeruginosa*) was isolated in two uterine samples, in one of them in high charge, and *Staphylococcus pseudintermedius* (*S. pseudintermedius*) in another one. Few colonies of *S. pseudintermedius* were also isolated in a meconium sample and very few colonies of *S. aureus* in the uterus of a bitch undergoing emergency CS (Table 3). Coagulase-negative Staphylococci were present in some amniotic and meconium samples. Also *Bacillus* spp. was frequently isolated, while *Micrococcus* spp., *Moraxella* spp., *Macrococcus* spp., *Glutamicibacter* spp., *Stenotrophomonas* spp. and *Psychrobacter* spp., were only occasionally identified (Table 3). Microorganisms belonging to the genus *Clostridium* or other anaerobic bacteria were never isolated (Table S1).

## 4. Discussion

Our data show that uterus and fetus are not sterile in healthy term canine pregnancies. If bacteria are isolated from placenta, fetal fluids and fetuses, colonization begins before birth and the newborn already harbours a bacterial community of maternal origin that influences health, development and further colonization at birth. This subject will still be debated because a limit of this and all previous investigations on different species, is that external contamination cannot be fully ruled out [2,4,7,8,9,10]. However, we worked in a surgical setting and tried to maintain sterility during all the sampling procedures.

The placenta represents a barrier to bacteria and one of its functions is to protect the fetus from microbial pathogens: Gram positive and negative intracellular bacteria have been documented in the basal plate of human placentae, both in pre-term and in term deliveries. Their role is uncertain: if viable, they could represent a reservoir that could initiate an inflammatory cascade and lead to adverse pregnancy outcomes such as preterm birth and chorioamnionitis [3]. Analogous findings were reported in cows, in which bacteria were observed in the endometrium and in the caruncolar stroma of slaughtered pregnant animals, of different pregnancy time, in the absence of inflammation [13].

Similar observations in dogs have been described in a study where bacteria were isolated from the fetal side of the placenta; however, the investigated puppies were delivered both vaginally and through CS [10] and the existence of a placental microbiota cannot be demonstrated in case of vaginal delivery and exposition to vaginal bacteria [4]. Besides, the work did not differentiate between elective and emergency CSs [10] and the uterus may be contaminated by bacteria of external origin if the cervix is open. In CSs performed because of dystocia, when the cervix is dilatated, bacteria were indeed isolated from the uterus of bitches after the extraction of all puppies and fetal membranes [14].

Our results add to existing literature on dogs because we exclusively investigated CS deliveries, with a separated analysis for elective and emergency surgeries, and we examined only the first extracted puppy, in order to minimize the possibility of contamination while avoiding to excessively slow down surgical and resuscitation procedures. Besides, we sampled the uterine side of the placenta.

A pioneering work by Harris et al. [15] stated the sterility of the human uterus on the basis of negative uterine cultures obtained from samples collected at CSs performed with intact membranes and closed or just opening cervix. In our work, the cervix was certainly open in emergency CSs, while we can suppose that it was closed or just opening in elective ones. A study by De Cramer and Nöthling [16] assessed the relationship between the decrease in plasma progesterone concentration and the time of cervical dilatation, and defined some cut-off values: bitches with a progesterone level below 2.7 ng/mL are expected to have a dilating cervix within 48 h while those with a progesterone level below 1 ng/mL within 24 h. The progesterone values when we preformed elective CSs were within the cited interval, but the authors themselves reported a large individual variation [16].

The hypothesis of a uterine microbiota is under discussion in humans and doubts about possible contaminations and methodological considerations have been raised [2]. In dogs, a microbial community was detected in the uterus during anaestrus, and described through metataxonomic investigations [17]. However, in works that use sequencing methods instead of microbial culture, it is impossible to demonstrate a community of living microorganisms with only the presence of bacterial DNA.

To our knowledge, isolation of bacteria from amniotic fluid samples of dogs has not been described before. In mares, bacteria were isolated in culture from the majority of amniotic fluid samples that were collected when the amniotic vesicle was clearly visible and that were aseptically aspirated into a syringe [18]. These and our findings are in contrast with recent investigations in humans, that failed to detect a bacterial community in amniotic fluid in uncomplicated term gestations using both culture and sequence-based methods [6] or sequence-technology exclusively [5]. However, amniotic fluid microbiota was found through sequencing in samples collected from the mouths of neonates as soon as they were born, both through CS and vaginal delivery [7].

Published data on bacterial findings from meconium of different species are hardly comparable with ours, because of different experimental conditions as we performed a rectal swab from a fetus delivered through CS, extracted from intact fetal membranes and immediately after resuscitation. Our data show a trend towards higher bacteria growth (more positive samples and higher growth in culture) from meconium samples when compared with the other locations. In the work by Zakošek Pipan et al. [10], puppy defecation was stimulated after first colostrum intake: this interval of time implies the possibility of colonization since bacterial colonization is rapid after birth [2]. The same critical factor of a time-lapse is present in some works in humans, in which the first-pass meconium was analyzed within a couple of hours from birth, detecting a meconium microbiota [7]; in other works, meconium was collected from diapers [19]. Bacterial communities were also detected in the meconium of vaginally delivered calves, immediately after birth [20]. However, the way of birth, either vaginal or through CS, gives a different imprint to the gut microbiota, which is dominated by species harboured in the mother’s vagina in the first case, and by skin commensals in the second [21].

Differently from the results of Zakošek Pipan et al. on dog placentae and meconium [10], we isolated various species of *Acinetobacter*, particularly *A. baumannii*, in almost half of the cultures. *Acinetobacter* spp. are ubiquitous bacteria that live in diverse environments such as soil or fresh water, and can also be isolated from dogs [22]. *A. baumannii* shows an extraordinary ability to accumulate antimicrobial resistance and is responsible of many nosocomial infections in humans. The species has also evolved in a veterinary nosocomial pathogen [22]. With the exception of *A. baumannii*, species identification in the genus *Acinetobacter* is not always possible without resorting to complex molecular biology methods, therefore we left *Acinetobacter* spp. if species identification was not certain through MALDI-TOF methodology.

We can confidently exclude the notion that the origin of our isolates is due to environmental contamination because they showed high growth in culture while the control swabs were negative, and especially because they come from two different surgical facilities where no case of nosocomial infection due to this bacterium had occurred.

In order to better understand the origin of the isolated bacteria, skin swabs or even swabs of the abdomen (in particular of the uterine surface) may be considered as further controls in future experiments.

*P. aeruginosa* is a ubiquitous bacterium that can be found in the environment, like in soil and in water. It is one of the major human opportunistic pathogens and it is a cause of diseases in both livestock and companion animals [23]. *S. pseudintermedius* belongs to the bacterial skin flora of canines and is a common opportunistic pathogen of dogs [24].

Other microbial genera that were isolated in low load, *Micrococcus* spp., *Dermacoccus* spp., *Macrococcus* spp., are Gram positive cocci, not easy to classify at species level with biochemical tests alone. They have been detected in some microbiological investigations of the air in both human [25] and veterinary hospital environments and found contaminating venous catheters [26]. As a common skin inhabitant, *Macrococcus* spp. is easily shed into the environment on desquamated epithelial skin cells [27]. It was also isolated from the skin of healthy dogs as well as from infection sites [28].

Also coagulase-negative Staphylococci (*S. equorum*, *S. hominis*, *S. saprophyticus*, *S. epidermidis*) occur very commonly as harmless commensals on human and animal skin, rectum and genitourinary tract.

Therefore, especially when we detected a low load, considering the presence on fur and desquamated epithelial cells, we cannot exclude that their isolation is due to contamination. Indeed, in case of cultures of human placenta sampled at CS that showed multiple isolates of *Propionibacterium* and *Staphylococcus* species [19], the criticism was that they could represent contaminant since they are ubiquitous normal skin commensals [2].

Genotype analysis could be useful to suggest whether a given bacterium is a contaminant or not: if the same genotype were found in various dams it would suggest an exogenous source.

Our work opens an interesting field of investigation and contributes to question the ‘sterile womb’ paradigm in dogs. The early establishment of a normal microbiome plays a key role in human and animal health; the symbiotic bacterial communities have a great importance in the immune system development and in the defense against infections. A slower growth rate was found in puppies that had no bacteria in their meconium or in the placenta [10].

Isolation of bacteria from placenta, fetal fluids and fetuses could prove that colonization begins before birth in dogs, and that the newborn already harbours bacterial strains of maternal origin that could have an influence on health, development and further colonization.

If colonization begins before birth in dogs, it is important to understand the factors, both maternal and environmental, that can affect the assembly of the microbiome. Further studies are necessary to confirm our observations and the evaluation of the cervix at the time of elective CSs could be useful to rule out vaginal colonization.

The technique that we used in this investigation, that is the traditional culture method, has the advantage to prove the viability of bacteria but the limit to fail to detect viable but non-cultivable microorganisms. The microbiota could be much richer than what we have observed, so that also metataxonomic investigations could help adding knowledge in this field.

## 5. Conclusions

We reported bacterial growth from samples obtained from healthy canine pregnancies at term, showing that the ‘womb’ may not be as sterile as previously thought. Our findings may contribute to pose a serious challenge to this paradigm and, even if further studies are needed to wholly understand the timing and mechanism of microbial colonization, they offer an interesting starting point for the advancement of the research in this fascinating and current topic.

## Figures and Tables

**Table 1:** Breed, age, weight and parity of the bitches included in the study.

Bitch ID	Breed	Age (yrs)	Weight (kg)	Primiparous	Elective C-Section	Dystocia	Surgical Facility	Litter	Progesterone
1	English Staffordshire Bull Terrier	6.8	13		X	-	1	5	2
2	English Staffordshire Bull Terrier	3.9	14		X	-	1	5	1.6
3	English Staffordshire Bull Terrier	3.1	15		X	-	1	6	1.5
4	Boston Terrier	2	4.5		X	-	1	6 + 1 immature macerated	*
5	Dogue de Bordeaux	3.2	62		X	-	2	9 + 1 macerated	2
6	English Staffordshire Bull Terrier	4.6	19		X	-	2	6	*
7	Dogue de Bordeaux	3.2	61		X	-	2	7	*
8	Dogue de Bordeaux	4.1	64		X	-	2	14 + 1 dead	5.4
9	Cavalier King Charles	3.3	12.4		X	-	2	6	*
10	Dogue de Bordeaux	3.9	55		X	-	2	9	1.9
11	Dachshund	8.2	9			Uterine inertia	1	3 (+1 dead naturally delivered)	/
12	Chihuahua	1.5	2.4	X		Fetal malposition	2	3	/
13	Newfoundland	4.1	65	X		Uterine inertia	2	8	/
14	Spitz	2.5	2.4	X		Uterine inertia	2	2	/
15	Chihuahua	1.10	3			Fetal malposition	2	1 (+2 naturally delivered)	/
	mean ± SD	3.7 ± 1.8	26.8 ± 25.9						

C-section was either planned or due to dystocic conditions. Surgery took place either at the University Veterinary Hospital of Turin (1) or at a private practice (2). The column ‘litter’ shows the number of newborns: when not differently specified, puppies were alive. The last column shows progesterone concentration at the moment of surgery; the symbol ‘*’ means that another sign was used to assess the end of pregnancy, that is the mucous plug passing the vulva.

**Table 2:** Positive cultures in the three locations (uterus, amniotic fluid, meconium), sampled when extracting the first fetus.

Bitch ID	Elective C-Section	Surgical Facility	Uterus (Placental Site)	Amniotic Fluid	Meconium
1	X	1	−−	−	−
2	X	1	/	−	−
3	X	1	+	+	+
4	X	1	+	+	+
5	X	2	−	+	+
6	X	2	+	+	+
7	X	2	+	−	+
8	X	2	+	+	+
9	X	2	−	−	+
10	X	2	+	−	+
11		1	/	−	−
12		2	−	+	+
13		2	+	−	+
14		2	−	−	+
15		2	+	−	+

No bacteria growth = —Missing samples = /Cesarean type (elective or emergency) and surgical facility (University Veterinary Hospital of Turin, 1 or private practice, 2) are reported in the second and third column.

**Table 3:** Bacteria isolated from the three locations: uterus (site of placental insertion), amniotic fluid and meconium of the first extracted fetus.

Bitch ID	Uterus	Bacteria	Growth	Amniotic Fluid	Bacteria	Growth	Meconium	Bacteria	Growth
3	+	*Micrococcus* spp. (*M.luteus*)	Low	+	*Acinetobacter* spp.	High	+	*Acinetobacter* spp.	High
		*Acinetobacter* spp.	High						
4	+	*Bacillus* spp.	High	+	*Bacillus* spp.	High	+	*Staphylococcus* spp. (*S.hominis*)	High
								*Acinetobacter baumannii*	High
5	−			+	*Staphylococcus equorum*	Moderate	+	*Staphylococcus equorum*	Low (1 CFU)
								Non-viable and unidentifiable strain (Gram positive cocci)	Moderate
6	+	*Dermacoccus nishinomiyaensis*	Low (2 CFU)	+	*Bacillus* spp. (*B.thuringiensis*)	High	+	*Staphylococcus saprophyticus*	Low (1 CFU)
		*Bacillus* spp.	Low					*Acinetobacter* spp.	High
7	+	*Pseudomonas aeruginosa*	High	−			+	*Bacillus* spp. (*B.cereus*)	Moderate
8	+	*Staphylococcus pseudintermedius*	High	+	*Glutamicibacter* spp. (*G.creatinolyticus*)	High	+	*Bacillus* spp.	High
9	−			−			+	*Stenotrophomonas* spp.	Low (3 CFU)
								*Acinetobacter* spp.	High
10	+	*Macrococcus* spp. (*M.canis*)	Low	−			+	*Acinetobacter baumannii*	High
		Unidentifiable strain	Low					*Bacillus* spp. (*B.cereus*)	High
12	−			+	*Moraxella* spp. (*M.osloensis*)	High	+	*Micrococcus* spp. (*M.luteus*)	Low *
					*Staphylococcus* spp. (*S.epidermidis*)	Low *			
13	+	*Pseudomonas aeruginosa*	Moderate	−			+	*Psychrobacter* spp. (*P.piechaudii*)	High
								*Staphylococcus pseudintermedius*	Low
								*Acinetobacter baumannii*	Moderate
14	−			−			+	*Acinetobacter baumannii*	High
15	+	*Staphylococcus aureus*	Low (1 CFU)	-			+	*Acinetobacter* spp.	High
		*Acinetobacter* spp.	Low *						

Growth in culture is reported (Low = 1–10 colony forming unit (CFU)/10 µL; Moderate11-30 CFU; High ≥31 CFU). The number of CFU appears in case of very low growth. ‘*’ = Bacterial cultures obtained from seeding of broth cultures.

## Data Availability

The data that are not contained in the article are available in supplementary material.

## Supplementary Materials

The following are available online at https://www.mdpi.com/article/10.3390/ani11051415/s1; Table S1: Animals bacteria resistances.
